# Traumatic brain injury extending to the striatum alters autonomic thermoregulation and hypothalamic monoamines in recovering rats

**DOI:** 10.3389/fnins.2023.1304440

**Published:** 2023-12-07

**Authors:** Antonio Verduzco-Mendoza, Daniel Mota-Rojas, Silvia Adriana Olmos Hernández, Arturo Gálvez-Rosas, Alexander Aguirre-Pérez, José Luis Cortes-Altamirano, Alfonso Alfaro-Rodríguez, Carmen Parra-Cid, Alberto Avila-Luna, Antonio Bueno-Nava

**Affiliations:** ^1^Programa de Doctorado en Ciencias Biológicas y de la Salud, Universidad Autónoma Metropolitana, Ciudad de México, Mexico; ^2^Neurofisiología, Conducta y Bienestar Animal, DPAA, Universidad Autónoma Metropolitana, Unidad Xochimilco, Ciudad de México, Mexico; ^3^Bioterio y Cirugía Experimental, Instituto Nacional de Rehabilitación LGII, SSa, Ciudad de México, Mexico; ^4^Neurociencias Básicas, Instituto Nacional de Rehabilitación Luis Guillermo Ibarra Ibarra (LGII), SSa, Ciudad de México, Mexico; ^5^Departamento de Quiropráctica, Universidad Estatal del Valle de Ecatepec, Ecatepec de Morelos, Estado de México, Mexico; ^6^Madrid College of Chiropractic, Real Centro Universitario Escorial María Cristina, Madrid, Spain; ^7^Unidad de Ingeniería de Tejidos, Instituto Nacional de Rehabilitación LGII, SSa, Ciudad de México, Mexico

**Keywords:** dopamine, noradrenaline, serotonin, hemorrhage, traumatic brain injury, core temperature, peripheral temperature, motor deficit

## Abstract

The brain cortex is the structure that is typically injured in traumatic brain injury (TBI) and is anatomically connected with other brain regions, including the striatum and hypothalamus, which are associated in part with motor function and the regulation of body temperature, respectively. We investigated whether a TBI extending to the striatum could affect peripheral and core temperatures as an indicator of autonomic thermoregulatory function. Moreover, it is unknown whether thermal modulation is accompanied by hypothalamic and cortical monoamine changes in rats with motor function recovery. The animals were allocated into three groups: the sham group (sham), a TBI group with a cortical contusion alone (TBI alone), and a TBI group with an injury extending to the dorsal striatum (TBI + striatal injury). Body temperature and motor deficits were evaluated for 20 days post-injury. On the 3rd and 20th days, rats were euthanized to measure the serotonin (5-HT), noradrenaline (NA), and dopamine (DA) levels using high-performance liquid chromatography (HPLC). We observed that TBI with an injury extending to the dorsal striatum increased core and peripheral temperatures. These changes were accompanied by a sustained motor deficit lasting for 14 days. Furthermore, there were notable increases in NA and 5-HT levels in the brain cortex and hypothalamus both 3 and 20 days after injury. In contrast, rats with TBI alone showed no changes in peripheral temperatures and achieved motor function recovery by the 7th day post-injury. In conclusion, our results suggest that TBI with an injury extending to the dorsal striatum elevates both core and peripheral temperatures, causing a delay in functional recovery and increasing hypothalamic monoamine levels. The aftereffects can be attributed to the injury site and changes to the autonomic thermoregulatory functions.

## Introduction

Brain injury is a global public health issue that can be caused by traumatic brain injury (TBI) or stroke and can even lead to death or disability (Guzik and Bushnell, 2017; Boursin et al., 2018; Haarbauer-Krupa et al., 2021; Saini et al., 2021; Giner et al., 2022). TBI patients exposed to high temperatures have a poor prognosis, which makes TBI patients with a febrile process or pyrexia more difficult to treat (Thompson et al., 2003; Gaither et al., 2015; Walter et al., 2016). Brain injury-induced hyperthermia can occur in patients with severe TBI and is associated with a poor prognosis (Thompson et al., 2007; Gaither et al., 2015; Atkins et al., 2017). Hyperthermia has been defined as an increase in body temperature, typically ranging from >37°C to >38.5°C (Geffroy et al., 2004; Rincon et al., 2014; Pegoli et al., 2020; Svedung Wettervik et al., 2021). In TBI patients, the incidence of hyperthermia is over 42% in the first 24 h (Rincon et al., 2014), with a prevalence of 68% to 85% during the acute phase (Svedung Wettervik et al., 2021).

Brain injury-induced hyperthermia above 39°C may have a multifactorial effect that includes thermoregulatory alterations in the hypothalamus, which is interconnected with brain areas such as the thalamus and the brain cortex (Risold et al., 1997; Saper, 2000; Thompson et al., 2003; Nomoto et al., 2004; Childs, 2008). The brain cortex is functionally interconnected with different brain regions, such as the striatum, pons, and thalamus, by afferent and efferent axons in the corticostriatal, corticopontine, corticothalamic, and thalamocortical pathways (Haber, 2016; Kremkow and Alonso, 2018; Rockland, 2019; Yahya, 2021; Verduzco-Mendoza et al., 2021b).

The striatum is part of the basal nuclei, and the dorsal striatum is associated with motor, oculomotor and executive/associative functions (Albin et al., 1989; Harting and Updyke, 2006; Liljeholm and O’doherty, 2012; Kandel et al., 2013). However, the role of the striatum in the process of thermoregulation is not yet fully understood. Parkinson’s disease (PD) is a pathology that involves the basal ganglia and autonomic dysfunction, leading in part to thermoregulatory disorders in patients (Coon and Low, 2018; McGregor and Nelson, 2019). PD originates from the progressive degeneration of the substantia nigra pars compacta, which projects specific axons to the dorsal striatum (Björklund and Dunnett, 2007; Surmeier et al., 2017; McGregor and Nelson, 2019). This degeneration results in reduced dopamine (DA) levels in the dorsal striatum and other structures, such as the brain cortex, leading to the presence of motor and nonmotor symptoms (McGregor and Nelson, 2019). A previous study proposed that the reduction in striatal DA synthesis and release could be linked to muscle thermogenesis (Gonzalez et al., 1986).

Hyperthermia induced by brain injury has been replicated in animal models of TBI and stroke (Thompson et al., 2005; Dietrich and Bramlett, 2007; de Jonge et al., 2018). In some studies, noninfectious hyperthermia was associated with changes in brain metabolism (Walter et al., 2016), intracranial pressure (Rossi et al., 2001), periventricular inflammation (Thompson et al., 2005), increased cerebral blood flow (Rossi et al., 2001; Mrozek et al., 2012) and severe injury (Thompson et al., 2003). Core temperature is indicative of the temperature of internal organs, such as the brain, which can be more difficult to measure (Hymczak et al., 2021). In patients with severe brain injury, cerebral core temperature can only be measured during surgical intervention (Rossi et al., 2001) and not in the postsurgical period. In clinical practice, temperature can be measured at central (pulmonary artery, urinary bladder, esophageal, rectal) and peripheral (body surface, oral, axilla, tympanic membrane, nasopharynx, temporal artery, forehead) sites (Niven et al., 2015; Hymczak et al., 2021). Peripheral measurements are less invasive, using infrared, electronic thermistors, chemical dots and glass thermometers (Niven et al., 2015). However, these methods of temperature measurement only use a dot as a reference.

The peripheral temperature is 4°C lower than the core temperature, but both temperatures are modulated by the thermoregulation system, which plays a vital role in homeostasis (Boulant, 2006; Skok et al., 2021). Previous studies have suggested that thermoregulation can be affected after brain injury (Gowda et al., 2018), which is explained by the interconnection between the hypothalamocortical pathway and the peripheral nervous system (Romanovsky, 2007). Hypothalamic monoamines are associated with thermoregulatory function (Feldberg and Myers, 1963; Cooper, 2002), and alterations in their levels may be an indicator of changes in hypothalamic thermoregulation. In the present study, we investigated whether TBI with an injury extending to the dorsal striatum alters peripheral and core temperatures as an indicator of autonomic thermoregulatory function. Additionally, we studied whether thermal modulation is accompanied by hypothalamic and cortical monoamine changes in rats with motor function recovery.

## Materials and methods

### Animals

Seventy-two male Wistar rats (280–310 g) were provided by Bioterio y Cirugía Experimental of Instituto Nacional de Rehabilitación LGII (INRLGII). All experimental protocols were approved by the Animal Care Committee of INRLGII (numbers 91/17, 06/18 and 82/21) and performed in accordance with the recommendations of the Official Mexican Standards (NOM-062-ZOO-1999, 2001). The animals were acclimatized to the laboratory environments at least 1 week prior to surgery and maintained on a 12-h light/12-h dark cycle, with food and water available *ad libitum*.

### Experimental design

**Figure 1:**
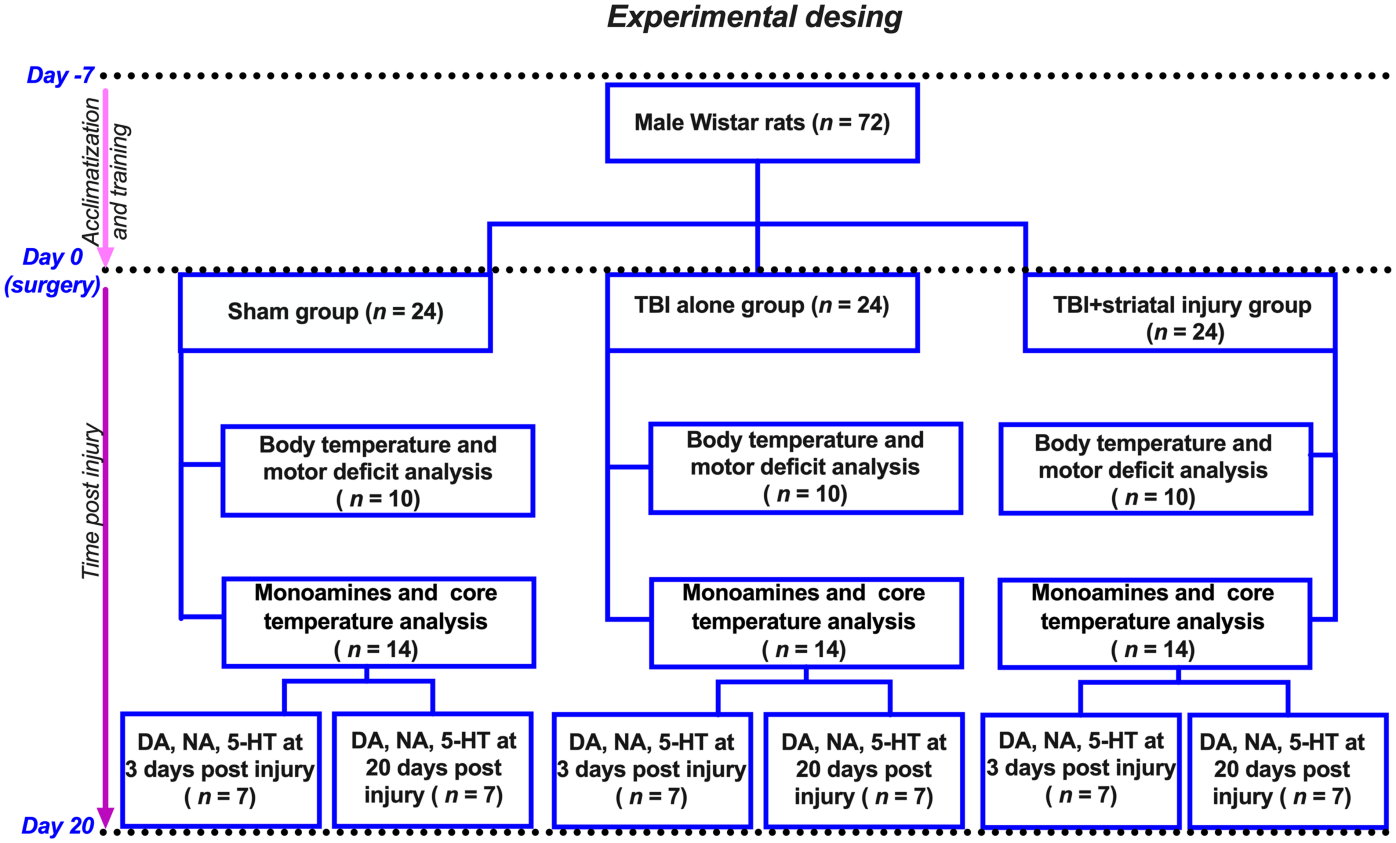
Flow diagram of experimental design. Seventy-two animals were divided into three groups: (1) Sham group (surgery, no injury); (2) TBI alone (cortical contusion); (3) TBI + striatal injury (cortical contusion with hemorrhagic extension). Hereof, 30 animals were utilized to measure body temperature and motor deficit analysis. The remaining 42 animals were used for monoamine analysis after 3 and 20 days post-injury, with 21 of them also used to measure core temperature in the external acoustic meatus.

Thirty animals were randomly assigned into the following three groups for the body temperature and motor deficit analysis (10 animals per group; see Figure 1): (1) the sham group, rats with surgery but without injury; (2) the TBI alone group, rats injured using cortical contusion procedures; and (3) the TBI + striatal injury group, animals with cortical contusion and hemorrhagic extension in the striatum.

Additionally, a total of 42 animals were used for the analysis of monoamines and measurement of the external acoustic meatus temperature (see Figure 1). These animals were assigned to three groups (sham, TBI alone and TBI + striatal injury), with 14 animals per group, 7 of which were sacrificed at 3 days post-injury and 20 days post-injury.

### Brain lesion procedures

All rats were anesthetized with a ketamine-xylazine mixture (80–10 mg/kg i.p.) and positioned in a stereotaxic frame (Stoelting Company, Wood Dale, IL, United States). The skull was exposed and trepanned (1 or 3 mm diameter) into the bone at the following coordinates (Paxinos and Watson, 2013): anteroposterior (AP) coordinates, +0.4 mm from bregma; lateral (L), −2.3 mm from midline; and vertical (V), 0, −2, and −4.5 mm below the dura mater for the sham, TBI alone and TBI + striatal injury groups, respectively. As stated above, the sham group was not injured. Following the surgical procedure, a recovery period of at least 24 h was provided for the rats before conducting the experiments.

The surgical differences for each brain lesion are described below.

#### Cortical TBI

The stereotaxic coordinates (AP and L) are described above. Brain injury was induced by impact using a stereotaxic impactor (Impact One, Leica Biosystems, Deer Park, IL, United States). The removable tip used was 2 mm in diameter, and a trephine hole of ~3 mm in diameter was used to position the impact accurately. The parameters used were as follows: impact velocity = 6 m/s; dwell time = 150 ms; and penetration depth = −2 mm below the dura mater. The parameters, derived from studies on the controlled cortical impact model, enable precise control of the injury’s force and velocity, in addition to the degree of tissue deformation, providing insight into biomechanical parameters associated with TBI (Edward Dixon et al., 1991; Osier N. and Dixon C. E., 2016; Osier N. D. and Dixon C. E., 2016).

#### TBI with striatal hemorrhagic injury

Using the same coordinates (AP and L), the animals underwent the cortical TBI procedure previously described. Additionally, in this group (TBI + striatal injury), an iron injection (2 μL of FeCl_2_, 50 mM; Sigma-Aldrich, St. Louis, MO) was localized in the dorsal striatum as a hemorrhagic extension after TBI. The depth was adjusted vertically (V) to −4.5 mm (below the dura mater). Combined cortical and striatal injury was performed according to the procedures outlined by Avila-Luna et al. (2018), Ramos-Languren et al. (2021), and García-Díaz et al. (2023). The volume and concentration of FeCl_2_ injected into the rat striatum in this study were directly related to the quantity of extravasated blood deposited within the brain following intracerebral hemorrhage in humans (Triggs and Willmore, 1984). According to Triggs and Willmore (1984), the equivalent dose of blood in the striatum was approximately 12 μL.

### Motor deficit analysis

The animals were videotaped and evaluated every 24 h for 20 days post-injury by using the beam-walking test previously validated by Brailowsky et al. (1986) and modified by Bueno-Nava et al. (2008) and Bueno-Nava et al. (2010). Motor deficits were evaluated using a scale from 0 to 6 according to the following criteria: 0 = animals without apparent deficits; 1 = widened base and four toes off the beam bilaterally; 2 = limp in one hind limb (hypotonus); 3 = at least 3 slips and/or 4 toes off the beam (unilaterally); 4 = falls or more than 3 slips; 5 = dragging a limb; and 6 = unable to run (Bueno-Nava et al., 2010). Scores were assigned in four different sections of the beam-walking test, for a maximum possible score of 24. The videotapes were reviewed by an investigator who was unaware of the treatment conditions.

### Temperature analysis

Animals were kept under temperature-controlled conditions at approximately 22°C with a humidity of approximately 55% (NOM-062-ZOO-1999, 2001). Images were obtained every 24 h for 20 days before the motor coordination test. The images were obtained using a digital infrared camera (ThermaCam E45, Flir Systems, Boston, MA, United States). A skin emissivity value of 0.98 W/m^2^ was employed. Each thermogram was obtained at a distance between the camera and animal body of 0.4–0.7 m. The camera was used with the following specifications: thermal resolution, 320 × 240; MSX resolution, 320 × 240; measurement accuracy, ±2°C; thermal sensitivity, <0.04°C; precision ±2°C or 2% of reading at room temperature of 10°C to 35°C; and image frequency, 60 Hz.

Each thermogram was analyzed using specialized software that was compatible with the camera (ThermaCam Researcher Basic 6.4 software, FLIR Systems, Wilsonville, OR, United States). The temperature of the animals was assessed in four specific regions of interest: head, positioned between the eyeball and the temporal bone, specifically near the external acoustic meatus; thoracic, anterior lateral flank located between the scapular region and the posterior diaphragm; abdominal, posterior lateral flank located between the diaphragm and the posterior extremity in the abdominal region; and tail, including proximal, medial and distal regions with respect to the rat body. The tail regions (proximal, medial, and distal) were identified using a previously validated method by El Bitar et al. (2014), which suggested that a proximal region should be placed 3 cm from the root of the tail, an intermediate region at the middle of the tail, and a distal region 3 cm from the tip of the tail. Additionally, the temperature of the external acoustic meatus was measured, as it closely approximates core temperature (Franklin et al., 1993; Bricknell, 1997).

### Analysis of catecholamine levels

The existing evidence suggests that neurotransmitters such as monoamines are altered during the acute phase (the initial hours or days) following a TBI, influencing functional recovery through a mechanism dependent on these neurotransmitters (Boyeson and Krobert, 1992; Bueno-Nava et al., 2008, 2010; Verduzco-Mendoza et al., 2021b).

The serotonin (5-HT), DA and noradrenaline (NA) levels were determined at 3 and 20 days post-injury using high-performance liquid chromatography (HPLC) as reported previously (Avila-Luna et al., 2016, 2018). Animals were decapitated, and the brain was rapidly removed and placed on an ice-cold plate to dissect the brain cortex and hypothalamus. In each rat, the brain cortex was separated on the ipsilateral and contralateral sides to the injury. Then, the tissues were homogenized (14,000 rpm at 4°C for 20 min) in 70% perchloric acid (HClO_4_; 94.6 mM) and sodium metabisulfite (5.26 mM) solution. The supernatant was filtered and injected into the HPLC system. The HPLC system was coupled to an amperometric electrochemical detector (model LC Epsilon (e-5), Bioanalytical Systems, Inc., West Lafayette, IN, United States) using a potential of +800 mV. Catecholamine contents were analyzed by interpolation using 5 standards with known concentrations (10–320 nM). Analytes were separated with an analytical column (Alltech, Adsorbosphere Catecholamine, 100 × 4.6 mm, 3 μm particle size). The mobile phase consisted of a phosphate buffer solution (29 mM, pH 3.1) containing sodium octyl sulfate (2.6 mM), EDTA (0.43 mM) and methanol (16.5% v/v). The flow rate was 1.6 mL/min, which was maintained using an HPLC pump (model LC-20 AD, Shimadzu Scientific Instruments, Inc., Maryland, United States). The peak signals were analyzed using ChromGraph software for Windows.

### Statistical analysis

The analysis of motor deficit scores was performed using the nonparametric Kruskal–Wallis test, followed by Dunn’s test. Values are expressed as medians ± interquartile range.

The statistical analyses of temperature and catecholamine levels were performed using one-way ANOVA followed by Tukey’s *post hoc* test. All values are expressed as the means ± standard errors of the mean (SEMs).

All statistical analyses were performed using the IBM Statistical Package for the Social Sciences (IBM SPSS) for Windows, version 22 (IBM Corp., Armonk, N.Y., United States). The statistical significance of these differences was set at *p* < 0.05.

## Results

### Motor coordination deficit

**Figure 2:**
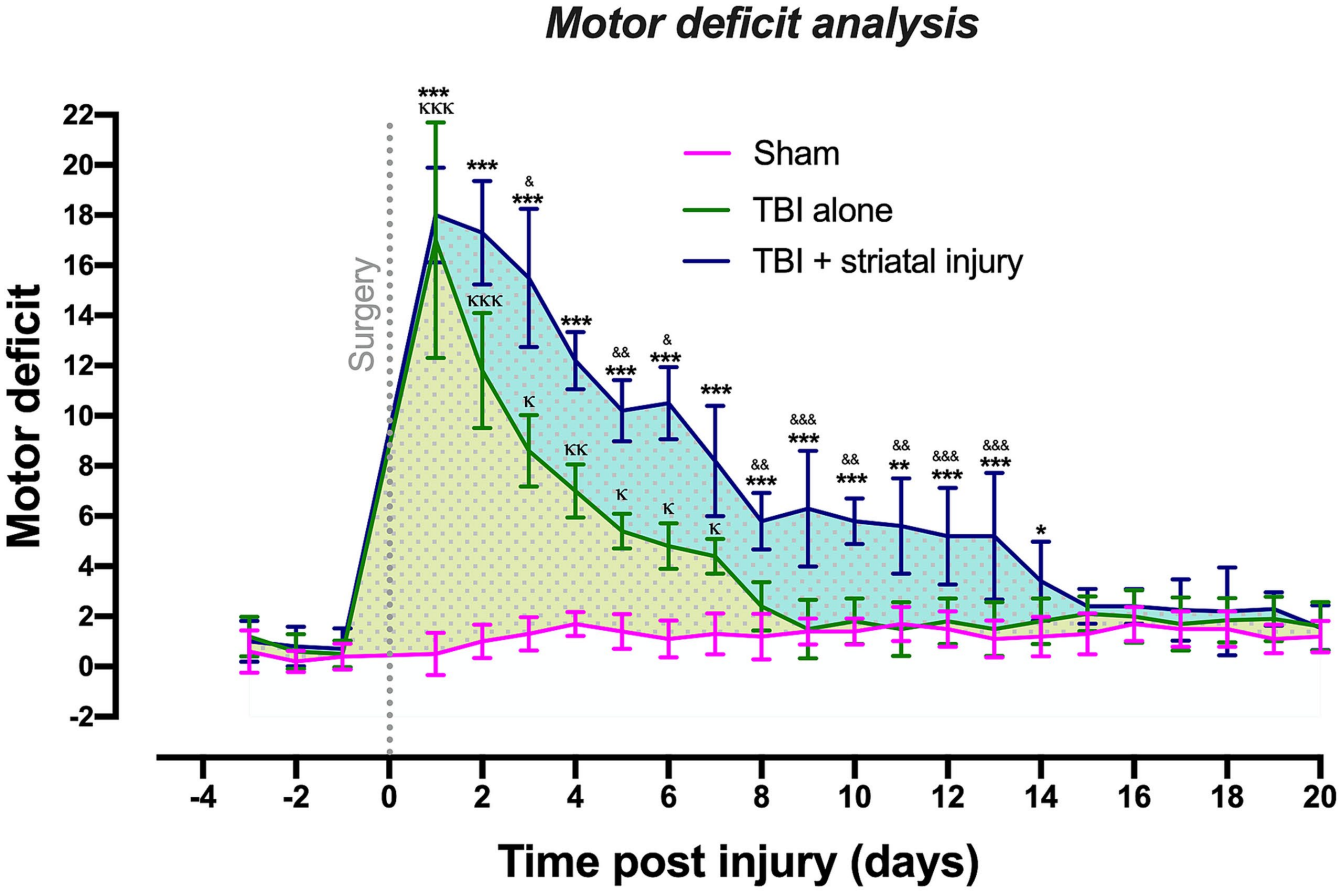
Scores obtained from the beam-walking test in the sham, TBI + striatal injury and TBI alone groups. The severity of motor coordination deficits in the TBI + striatal injury group increased from Day 1 to Day 14. The TBI alone group showed an increase from Day 1 to Day 7. Values are expressed as medians ± interquartile ranges. The statistical analysis of motor deficits was performed with the nonparametric Kruskal–Wallis test, followed by Dunn’s multiple comparisons test. ^***^p < 0.001, ^**^p < 0.01, ^*^p < 0.05: TBI + striatal injury group vs. the sham group. ^&&&^*p* < 0.001, ^&&^*p* < 0.01, and ^&^*p* < 0.001: TBI + striatal injury group vs. TBI alone group. ^ΚΚΚ^*p* < 0.001, ^ΚΚ^*p* < 0.01, ^Κ^*p* < 0.05: TBI alone group vs. the sham group. The blue and yellow dotted patterns represent the first temporal window associated with motor deficit period. The second window was represented by the motor recovery period, which showed a small, dotted area and without statistical differences.

Figure 2 shows that TBI alone significantly increased the motor coordination deficit compared with that in the sham group from Day 1 to Day 7 post-injury. Recovery was observed at 8 days post-injury, and it remained constant for 12 days after the motor deficit period. The TBI alone and TBI + striatal injury groups exhibited significantly increased motor coordination deficits compared with the sham group. In the TBI + striatal injury group, the motor deficit decreased to normal levels on Day 15 post-injury and was sustained for the remaining 6 days. These results confirmed that the TBI + striatal injury group had more severe deficits than the TBI alone group.

On the first day post-injury, motor coordination deficits were observed in all lesioned groups, with maximum scores of 5 for some sections of the elevated wooden beam. The TBI alone and TBI + striatal injury groups exhibited the highest motor deficit scores compared to the sham group, in which motor effects were not observed in any animal, with scores between 0 and 2 in most evaluated sections.

For the TBI alone group, the beam walking test revealed two temporal windows: the first was an injury period of 7 days (Figure 2, yellow dotted pattern), and the second was a period with consistent recovery of 13 days, which is shown as a small dotted area with no statistical differences. The TBI + striatal injury group exhibited an injury period between 1 and 14 days post-injury (Figure 2, blue dotted pattern), with a consistent motor recovery period of 6 days.

### Head, thorax, abdomen and tail temperature analysis

#### Head temperature

**Figure 3:**
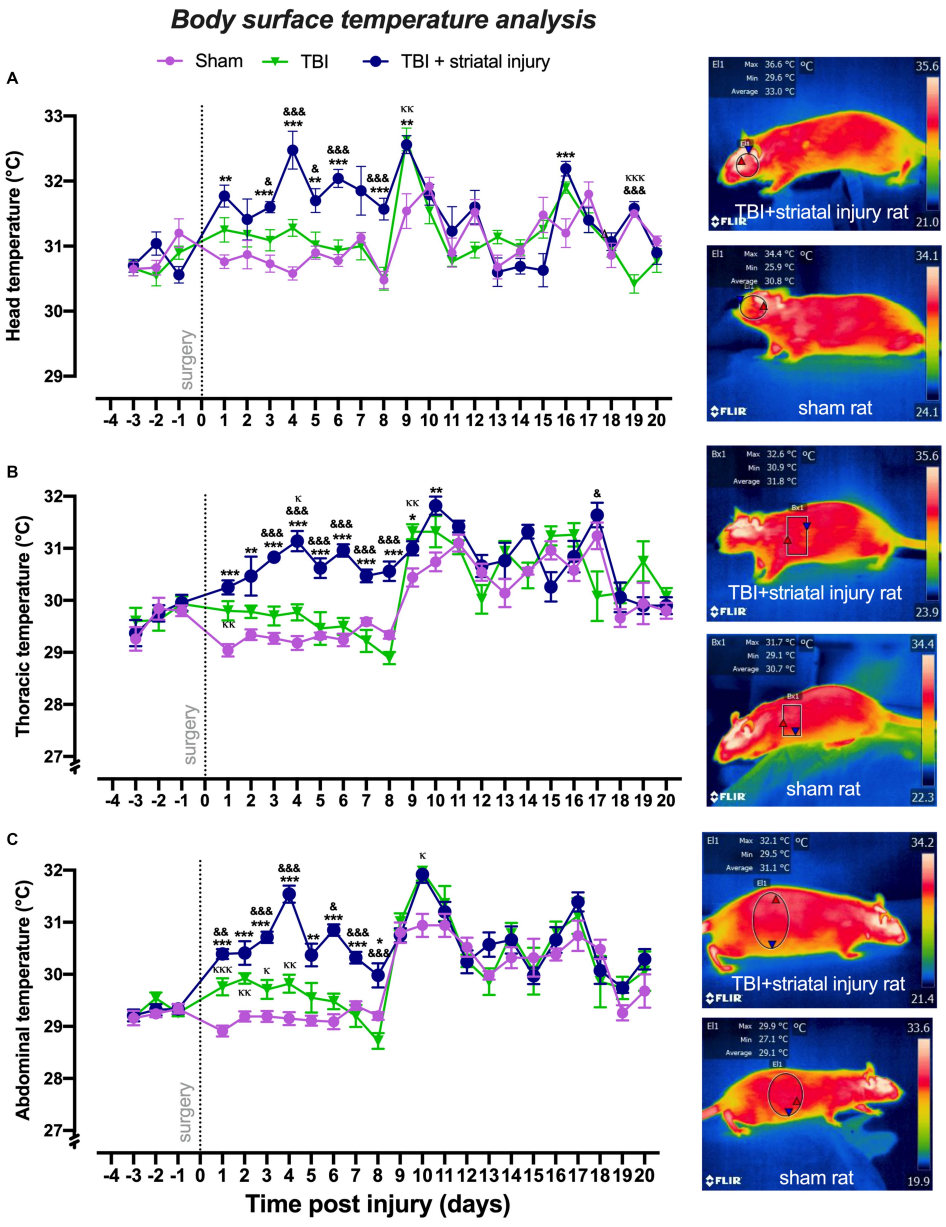
Peripheral temperatures obtained by infrared thermography measurement in the head **(A)**, thorax **(B)**, and abdomen **(C)** post-injury. The TBI + striatal injury group showed increased peripheral temperatures. A representative thermogram is presented, illustrating the temperatures in a sham rat and a rat with TBI + striatal injury. Values are expressed as the means ± standard errors (SEM). The statistical analysis was performed with one-way ANOVA followed by Tukey’s test to compare the means between the groups. ^***^p < 0.001, ^**^p < 0.01, ^*^p < 0.05: TBI + striatal injury group vs. the sham group. ^&&&^*p* < 0.001, ^&&^*p* < 0.01, and ^&^*p* < 0.001: TBI + striatal injury group vs. TBI alone group. ^ΚΚΚ^*p* < 0.001, ^ΚΚ^*p* < 0.01, ^Κ^*p* < 0.05: TBI alone group vs. the sham group.

As shown in Figure 3A, in the motor deficit period, the TBI + striatal injury group had significantly increased (*p* < 0.05, Tukey’s test) head temperature values at Days 1, 3–6, 8 and 9 compared with the sham group. In this same period, the TBI alone group showed significant differences (*p* < 0.05, Tukey’s test) in head temperature values at Days 3–6 and 8 compared with the TBI + striatal injury group (Figure 3A) but not with the sham group.

#### Thorax temperature

In the motor deficit period, increases (*p* < 0.05, Tukey’s test) in thoracic temperature values were observed in the TBI + striatal injury group from Day 1 to Day 10 compared with the sham group (Figure 3B).

The TBI alone group showed significant differences (*p* < 0.05, Tukey’s test) in the thoracic temperature values from Day 3 to Day 8 (compared with the TBI + striatal injury group; Figure 3B).

#### Abdominal temperature

As shown in Figure 3C, in comparison with the sham group, the TBI + striatal injury group exhibited significantly increased (*p* < 0.05, Tukey’s test) abdominal temperatures from Day 1 to Day 8. In this same period, the TBI alone group showed significant differences (*p* < 0.05, Tukey’s test) in abdominal temperatures from Day 1 to Day 8 compared with the TBI + striatal injury group (Figure 3C), except for the second day, which did not show significant changes (*p* > 0.05, Tukey’s test). In comparison with the sham group, the temperatures in the TBI alone group significantly increased from Day 1 to Day 4. However, in the recovery period, this effect disappeared among the injury groups.

#### Tail temperature

**Figure 4:**
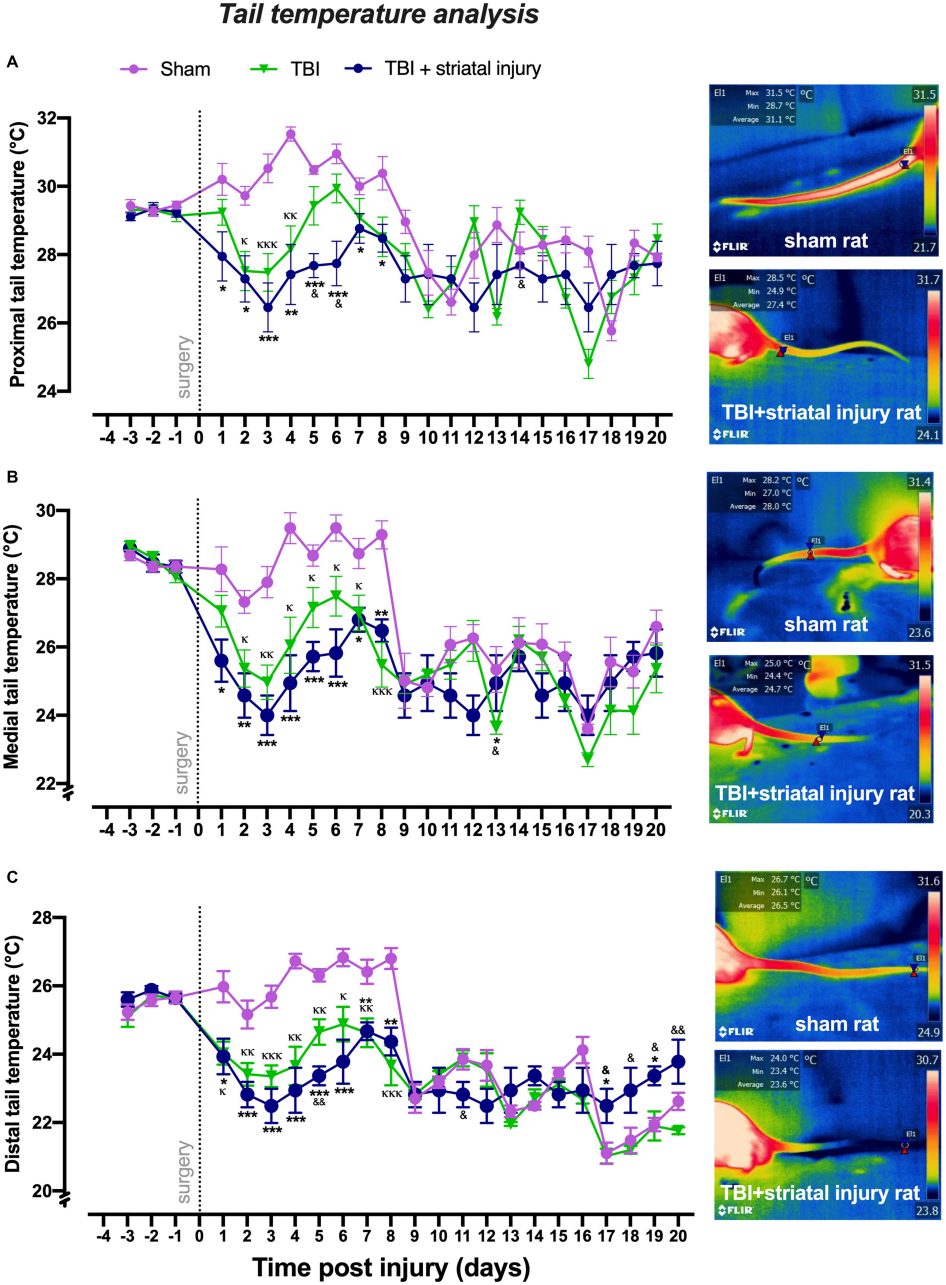
Peripheral temperatures obtained by infrared thermography measurement in the proximal **(A)**, medial **(B)**, and distal **(C)** sections of the tail following injury. The TBI + striatal injury group exhibited decreased peripheral temperature values. A representative thermogram is shown, displaying temperatures from a sham rat and a rat with TBI + striatal injury. Values are expressed as the means ± standard errors (SEM). The statistical analysis was performed with one-way ANOVA followed by Tukey’s test to compare the means between the groups. ^***^p < 0.001, ^**^p < 0.01, ^*^p < 0.05: TBI + striatal injury group vs. the sham group. ^&&^*p* < 0.01 and ^&^*p* < 0.001: TBI + striatal injury group vs. the TBI alone group. ^ΚΚΚ^*p* < 0.001, ^ΚΚ^*p* < 0.01, ^Κ^*p* < 0.05: TBI alone group vs. the sham group.

As shown in Figures 4A–C, compared to the sham group, the TBI + striatal injury group exhibited a significant decrease (*p* < 0.05, Tukey’s test) in tail temperatures at the proximal, medial, and distal regions from Day 1 to Day 8. The TBI alone group exhibited a significant decrease (*p* < 0.05, Tukey’s test) in proximal (from Day 2 to Day 4), medial (from Day 2 to Day 8) and distal (from Day 1 to Day 8) tail temperatures compared with the sham group. In both the TBI alone and TBI + striatal injury groups, the effect of reduced temperature remained consistent during the motor deficit period. In the recovery period, however, this effect disappeared, except in the distal tail temperature, which showed an increase in the last 4 days of the study.

### External acoustic meatus temperature analysis

**Figure 5:**
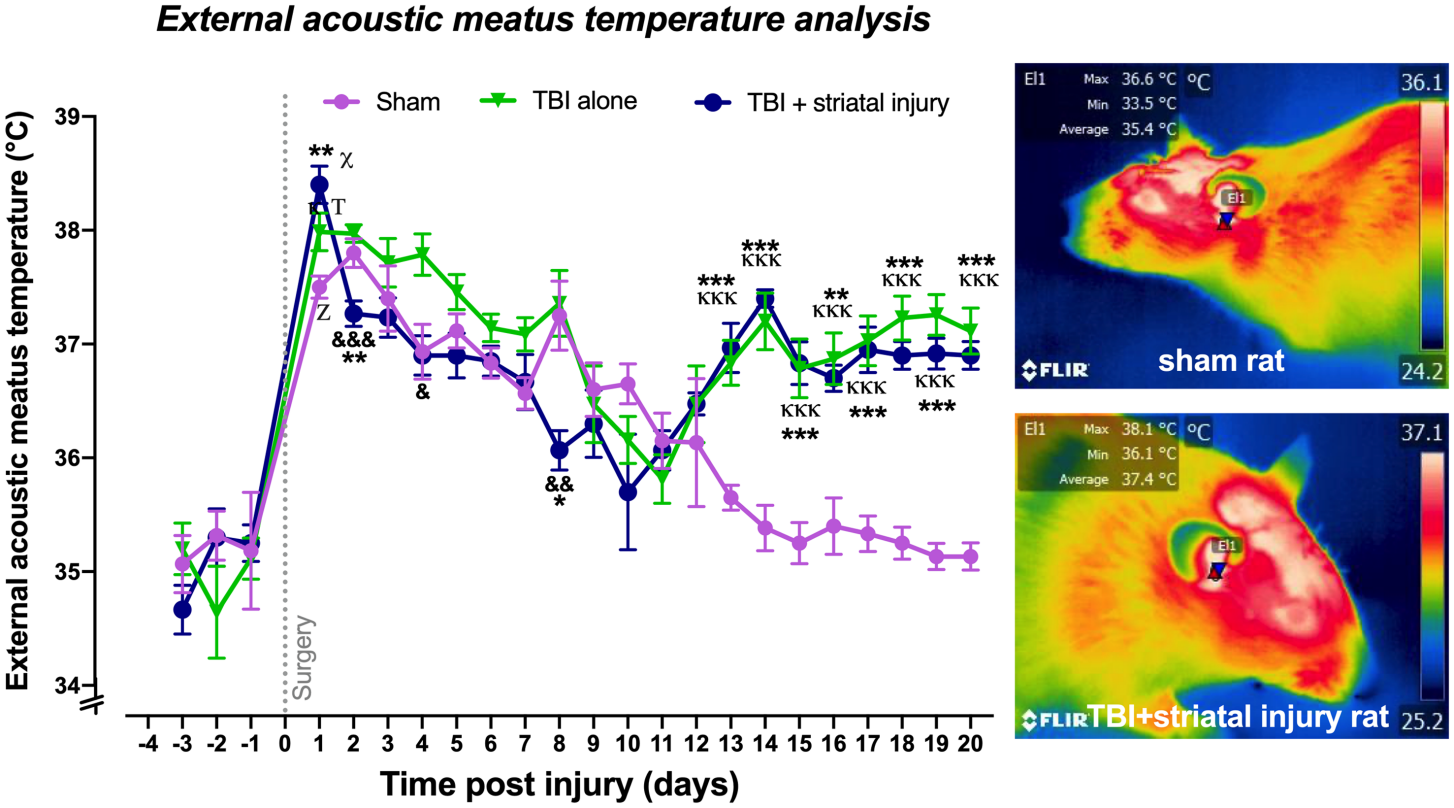
Core temperatures obtained by infrared thermography measurement in the external acoustic meatus post-injury. The temperature of the external acoustic meatus was measured as an approximation of the core temperature. A representative thermogram is shown, displaying the temperatures of a sham rat and a rat with TBI + striatal injury. Values are expressed as the means ± standard errors (SEM). The statistical analysis was performed with one-way ANOVA followed by Tukey’s test to compare the means between the groups. ^*x*,Τ,*z*^*p* < 0.001 for the TBI with striatal injury, TBI alone, and sham groups, respectively, vs. their respective basal values. ^***^p < 0.001, ^**^p < 0.01, ^*^p < 0.05: TBI + striatal injury group vs. the sham group. ^&&&^*p* < 0.001, ^&&^*p* < 0.01, and ^&^*p* < 0.001: TBI + striatal injury group vs. TBI alone group. ^ΚΚΚ^*p* < 0.001, ^Κ^*p* < 0.05: TBI alone group vs. the sham group.

As described in the Materials and Methods section, the temperature of the external acoustic meatus was taken as an approximation of the core temperature. As shown in Figure 5, the external acoustic meatus temperatures on Day 1 post-injury were significantly increased in the sham, TBI alone, and TBI + striatal injury groups by approximately 2.3 (*p* < 0.001, Tukey’s test), 2.9 (*p* < 0.001, Tukey’s test) and 3.1°C (*p* < 0.001, Tukey’s test), respectively, compared with the respective values on Day −1 preinjury. The TBI alone (*p* = 0.047, Tukey’s test) and TBI + striatal injury (*p* < 0.001, Tukey’s test) groups exhibited significantly increased external acoustic meatus temperatures on Day 1 compared with the sham group. Both the TBI alone and TBI + striatal injury groups showed an increase in external acoustic meatus temperatures from Day 13 (TBI alone group, *p* < 0.001, Tukey’s test; TBI + striatal injury group, *p* < 0.001, Tukey’s test) to Day 20 (TBI alone group, *p* < 0.001, Tukey’s test; TBI + striatal injury, *p* < 0.001, Tukey’s test) post-injury compared with the sham group (Figure 5).

### NA, DA and 5-HT levels

#### Cortical monoamine levels

**Figure 6:**
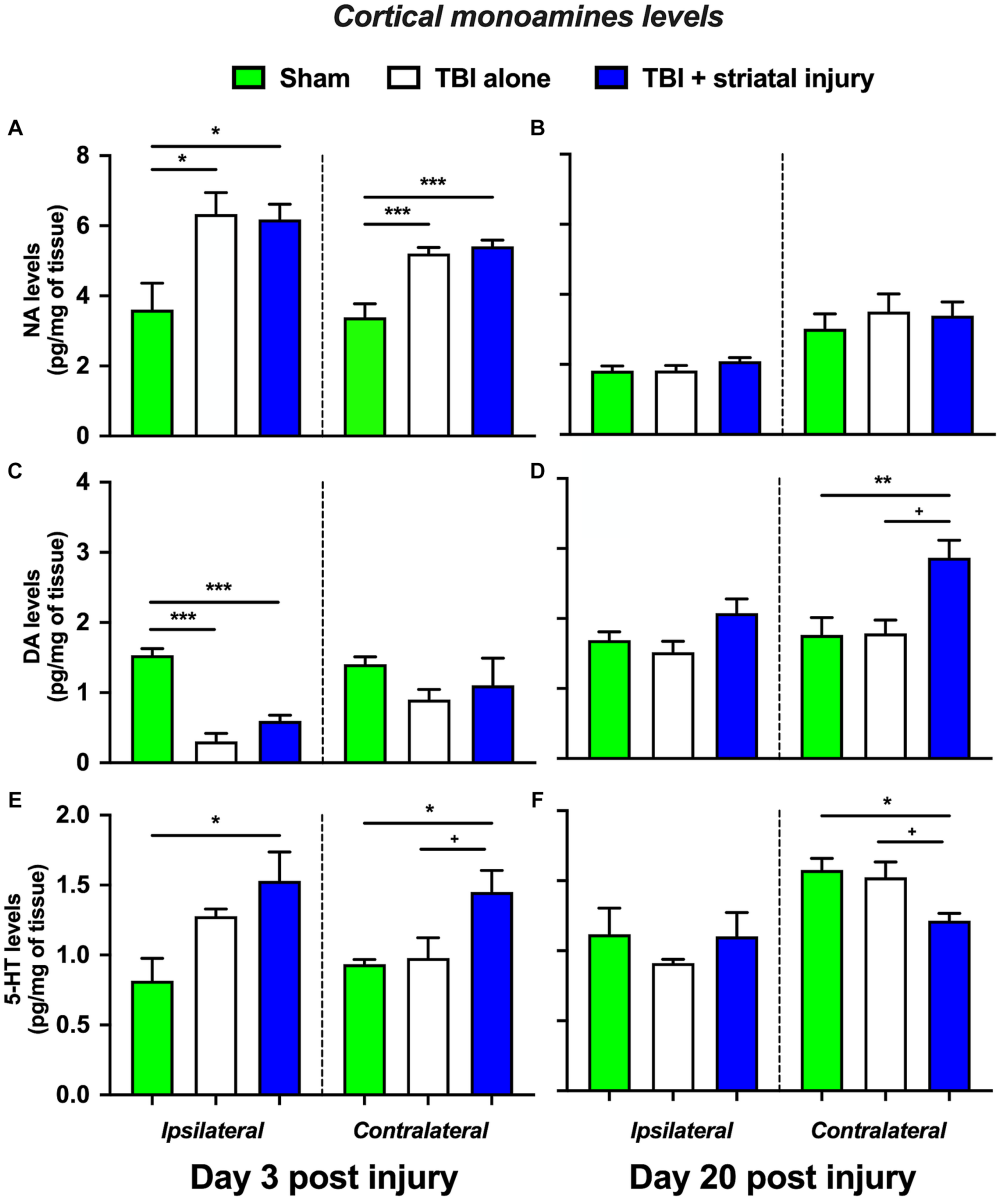
NA **(A-B)**, DA **(C-D)**, and 5-HT **(E-F)** levels in the brain cortex ipsilateral and contralateral to the lesion at 3 and 20 days post-injury. Values are expressed as the means ± standard errors (SEM). The statistical analysis was performed with one-way ANOVA followed by Tukey’s test to compare the means between the groups. ^***^p < 0.001, ^**^p < 0.01, and ^*^p < 0.05 vs. the sham group. ^+^*p* < 0.05 vs. the TBI without striatal injury group.

Cortical NA, DA and 5-HT levels are shown in Figures 6A–F. The TBI and TBI + striatal injury groups showed a significant increase in NA levels in the brain cortex ipsilateral to the injury at 3 days post-injury of 6.33 ± 0.61 (*p* = 0.015, Tukey’s test) and 6.18 ± 0.43 pg./mg of tissue (*p* = 0.022, Tukey’s test), respectively, compared with the sham group (3.60 ± 0.76 pg./mg of tissue; Figure 6A). This increase was also observed on the contralateral side for both the TBI and TBI + striatal injury groups at 3 days post-injury, with increase of 5.21 ± 0.17 (*p* < 0.001, Tukey’s test) and 5.41 ± 0.17 pg./mg of tissue (*p* < 0.001, Tukey’s test), respectively, compared with the sham group (3.39 ± 0.38 pg./mg of tissue; Figure 6A).

A significant decrease in DA levels was observed in the ipsilateral brain cortex at 3 days post-injury in both the TBI alone and TBI + striatal injury groups of 0.30 ± 0.11 pg./mg of tissue (*p* < 0.001, Tukey’s test) and 0.60 ± 0.08 pg./mg of tissue (*p* < 0.001, Tukey’s test), respectively, compared with the sham group (1.50 ± 0.09 pg./mg of tissue; Figure 6C). On the contralateral side, the TBI + striatal injury group showed a significant increase in DA levels of 2.87 ± 0.25 pg./mg of tissue at 20 days post-injury compared with the sham (1.76 ± 0.25 pg./mg of tissue; *p* = 0.009, Tukey’s test) and TBI alone (1.79 ± 0.19 pg./mg of tissue*; p* = 0.011, Tukey’s test) groups, respectively (Figure 6D).

In the TBI + striatal injury group, a significant increase was observed in 5-HT levels in both the ipsilateral and contralateral cortical sides on Day 3 post-injury of 1.53 ± 0.21 (*p* = 0.011, Tukey’s test) and 1.45 ± 0.15 pg./mg of tissue (*p* = 0.045, Tukey’s test), respectively, compared with the sham group (ipsilateral, 0.82 ± 0.16 pg./g of tissue; contralateral 0.93 ± 0.03 pg./mg of tissue; Figure 6E). At 20 days post-injury, a significant decrease was observed in 5-HT levels on the contralateral side of the brain cortex of 1.21 ± 0.05 pg./mg of tissue (*p* = 0.019, Tukey’s test) compared with the sham group (1.58 ± 0.08 pg./mg of tissue; Figure 6F).

#### Hypothalamic monoamine levels

**Figure 7:**
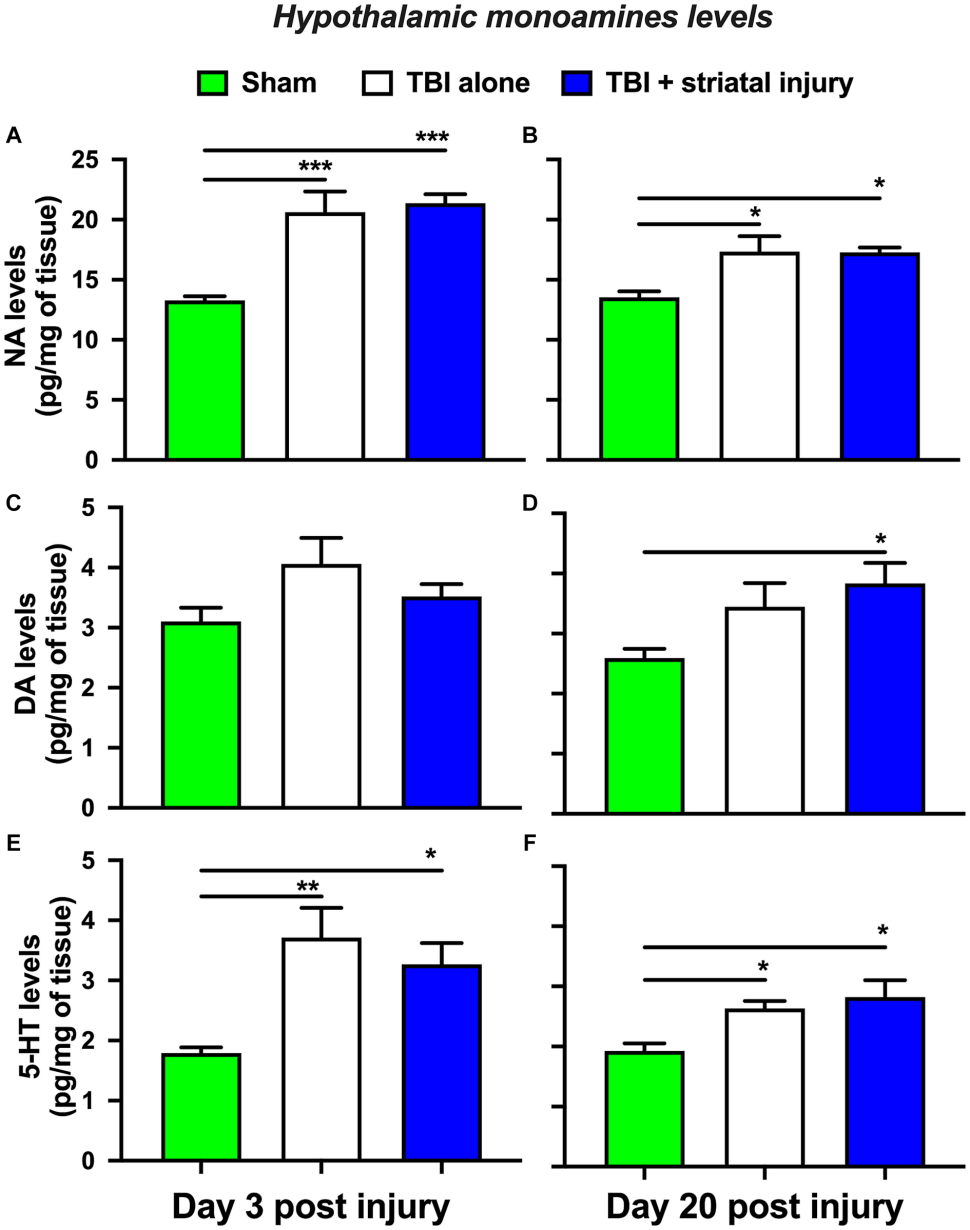
NA **(A-B)**, DA **(C-D)**, and 5-HT **(E-F)** levels in the hypothalamus at 3 and 20 days post-injury. Values are expressed as the means ± standard errors (SEM). The statistical analysis was performed with one-way ANOVA followed by Tukey’s test to compare the means between the groups. ^***^p < 0.001, ^**^p < 0.01, and ^*^p < 0.05 vs. the sham group.

The TBI alone group showed a significant increase in NA levels in the hypothalamus at 3 and 20 days post-injury of 20.61 ± 1.73 (*p* < 0.001, Tukey’s test; Figure 7A) and 17.33 ± 1.28 pg./mg of tissue (*p* = 0.012, Tukey’s test; Figure 7B), respectively, compared with the sham group, which had levels of 13.28 ± 0.34 and 13.54 ± 0.49 pg./mg of tissue at 3 and 20 days post-injury, respectively. This increase was also observed in the TBI + striatal injury group at 3 and 20 days post-injury, by 21.35 ± 0.74 (*p* < 0.001, Tukey’s test; Figure 7A) and 17.28 ± 0.40 pg./mg of tissue (*p* = 0.013, Tukey’s test; Figure 7B), respectively, compared with the respective sham group.

As shown in Figure 7D, the TBI + striatal injury group exhibited a significant increase in DA levels of 3.83 ± 0.34 pg./mg of tissue (*p* = 0.023, Tukey’s test) at 20 days post-injury compared with the sham group (2.59 ± 0.15 pg./mg of tissue). The TBI alone group showed a significant increase in hypothalamic 5-HT levels at 3 and 20 days post-injury of 3.71 ± 0.49 (*p* = 0.003, Tukey’s test; Figure 7E) and 2.63 ± 0.12 pg./mg of tissue (*p* = 0.045, Tukey’s test; Figure 7F), respectively, compared with the sham group, which had levels of 1.79 ± 0.09 and 1.92 ± 0.13 pg./mg of tissue at 3 and 20 days post-injury, respectively. This increase was also observed in the TBI + striatal injury group at 3 and 20 days post-injury, by 3.27 ± 0.35 (*p* = 0.022, Tukey’s test; Figure 7E) and 2.82 ± 0.28 pg./mg of tissue (*p* = 0.011, Tukey’s test; Figure 7F), respectively, compared with the respective sham group.

## Discussion

In the present study, we demonstrated that TBI with striatal hemorrhagic extension (TBI + striatal injury group) alters peripheral and core temperatures, which are indicators of autonomic thermoregulatory function (Fealey, 2013; Nakamura, 2018; Morrison and Nakamura, 2019). Additionally, thermal modulation was accompanied by changes in hypothalamic and cortical monoamine levels, which occurred during motor deficits and motor function recovery. In both periods, motor deficits and motor recovery in the rats with TBI alone or TBI + striatal injury increased hypothalamic NA and 5-HT levels. Furthermore, NA and 5-HT levels were increased in the brain cortex at 3 and 20 days post-injury.

In the TBI alone group, the motor deficit test showed two temporal windows: an initial 7-day injury period followed by a 13-day recovery period. Both motor deficit and motor recovery periods were demonstrated in our previous studies with the use of cortical ablation and cortical hemorrhagic injury (Bueno-Nava et al., 2008, 2010; Avila-Luna et al., 2018; Gálvez-Rosas et al., 2019; Ramos-Languren et al., 2021). These results were replicated in both the TBI alone and TBI + striatal injury groups, although the TBI + striatal injury led to a motor deficit lasting 14 days compared with the 7 days observed in the TBI alone group. Furthermore, the TBI + striatal injury group exhibited a higher severity than the TBI alone group, confirming the greater impact of the combined condition (Avila-Luna et al., 2018). The striatum receives afferent input from the cerebral cortex, including the areas associated with cognitive functions, sensory processing, and motor commands (Graybiel and Grafton, 2015; Bolam and Ellender, 2016; Grillner and Robertson, 2016; Haber, 2016). Concurrently, the basal ganglia output nuclei (internal segment of the globus pallidus and substantia nigra pars reticulata) send efferent outputs to the thalamus, which is a deep-seated brain structure that acts as a relay center for sensory and motor signals (Bolam and Ellender, 2016; Haber, 2016). Additionally, the striatum receives afferent input from the thalamus, which differs from cortical inputs in its synaptic contacts with striatal neurons (Ding et al., 2008). According to our results, the intrinsic relationship between the striatum, cerebral cortex, and thalamus is crucial for motor coordination, and corticostriatal injury can disrupt this balance, highlighting the significance of this interconnection in normal motor function (Costa et al., 2006; Bueno-Nava et al., 2010; Avila-Luna et al., 2018).

Previous studies have shown that posttraumatic hyperthermia develops after a severe brain injury (Rossi et al., 2001; Thompson et al., 2003, 2005; Gaither et al., 2015; Zakhary and Sabry, 2017). The cerebral core temperature, measured with a thermistor, showed an increase after severe injury (Rossi et al., 2001). Rossi et al. (2001) reported that the temperature in the brain is dependent on local metabolism, cerebral blood flow, and the temperature of the perfused blood. In brain injury, hyperthermia can originate from damage in the hypothalamic thermoregulatory center (Zakhary and Sabry, 2017; Bao et al., 2021). Interestingly, our results demonstrated that TBI and striatal injury increased head, thoracic and abdominal temperatures in the TBI + striatal injury group. However, both the TBI alone and TBI + striatal injury groups showed a significant decrease in distal, medial and proximal tail temperatures compared with the sham group. In rats, the tail occupies 9% of the body area and has the capacity to dissipate only 17% of the body heat (Rand et al., 1965; Gouma et al., 2012; Škop et al., 2020). This could explain why the thermoregulatory capacity of the rat’s tail was not sufficient to dissipate heat from the body surface in the TBI + striatal injury group (Škop et al., 2020).

An explanation for the increase in temperature in the sham and TBI groups between days 8 and 9 post-injury may be related to a variation in the ambient temperature. It is well known that small-scale temperature variation can play an important role in species’ thermal adaptation and can magnify the effects of increasing temperatures (Mota-Rojas et al., 2021; Verduzco-Mendoza et al., 2021a; Laird-Hopkins et al., 2023). It is possible that the temperature increase observed in both the injured and sham groups during this period was a response to ambient temperature variation, a result of thermal adaptation (Mota-Rojas et al., 2021). This may explain the decreased tail temperatures observed during the same timeframe, possibly due to vasoconstriction in response to the temperature variation (Verduzco-Mendoza et al., 2021a). The amplified response in the sham group could be attributed to the fact that they are uninjured subjects (Lezama-García et al., 2022).

Further studies are required to establish the relationship between cerebral and body temperatures after brain injury. In our results, the temperature of the external acoustic meatus closely matched the core temperature (Franklin et al., 1993; Bricknell, 1997). This core temperature increased during motor deficits on Day 1 post-injury in both the TBI-alone and TBI + striatal injury groups compared to the sham group. Subsequently, these groups displayed a trend of decreasing temperature. However, this reduction did not occur in both injured groups, as they continued to exhibit an elevated core temperature during the motor recovery period. In contrast, the core temperatures were reverted to baseline levels in the sham group. This hyperthermia could possibly stem from temperature dysregulation, an indicator of autonomic dysfunction (Takahashi et al., 2015). One hypothesis to explain the sustained hyperthermia from Day 13 to Day 20 post-injury in the injured groups is that it may result from vasospasm. This hypothesis is supported by observations in patients with subarachnoid hemorrhage (SAH), who exhibit non-infectious hyperthermia that persists for several days following the vascular event. This condition has been linked to the development of both vasospasm and symptomatic vasospasm (Rabinstein and Sandhu, 2007). Such non-infectious refractory hyperthermia could also be related to vascular vasospasm, potentially providing a parallel between core and peripheral temperature patterns observed in our study. It is known that vasospasm induces a concomitant increase in cerebral blood flow (CBF) velocity in the brain’s principal arteries (Carr et al., 2013).

The second hypothesis to explain the fluctuations in core and peripheral temperatures can be attributed to severe injury in the injured groups. This severity can be understood by considering the interconnection between the injury site and thermoregulatory centers along the corticohypothalamic, hypothalamocortical, cortex-basal ganglia-hypothalamus pathways (Risold et al., 1997; Saper, 2000; Romanovsky, 2007; Childs, 2008; Saper and Lowell, 2014; Bolam and Ellender, 2016). Additionally, the involvement of the striatum in the basal ganglia provides a further explanation for our findings (Feldman, 1966; Graybiel and Ragsdale, 1979; Risold et al., 1997; Bolam and Ellender, 2016).

The striatum, a key structure within the basal ganglia, is central to numerous brain functions, such as movement, reward, and cognition (Liljeholm and O’doherty, 2012; Kandel et al., 2013). The striatum has an intricate relationship with thermoregulation through direct and indirect connections with the hypothalamus, the primary thermoregulatory center in the brain (Feldman, 1966; Bolam and Ellender, 2016; Kullmann and Veit, 2021). Moreover, the striatum’s involvement in neurotransmitter release, especially dopamine, plays a pivotal role in thermoregulatory processes (Gonzalez et al., 1986; Björklund and Dunnett, 2007;Shin et al., 2011; Jenkins et al., 2018). Given its functions in motor response generation and sensory integration, the striatum might influence behaviors aimed at temperature regulation (Gonzalez et al., 1986). Additionally, diseases affecting the striatum, such as Parkinson’s disease, often exhibit thermoregulatory abnormalities (Coon and Low, 2018; McGregor and Nelson, 2019).

The hypothalamus establishes reciprocal interconnections with the brain cortex, including the medial prefrontal cortex (Kremkow and Alonso, 2018; Hymczak et al., 2021), through the hypothalamocortical and corticohypothalamic pathways. The indirect interconnection between the hypothalamus and brain cortex (Risold et al., 1997) by noradrenergic, dopaminergic and serotonergic projections has been studied (Heym and Gladfelter, 1982; Saper, 2000; Schwarz and Luo, 2015; Jenkins et al., 2016; Reppucci and Petrovich, 2016; Vitrac and Benoit-Marand, 2017; Jenkins et al., 2018). Hypothalamic monoamines are associated with thermoregulatory function (Cooper, 2002; Childs, 2008), and their alteration may be an indicator of changes at the hypothalamic level.

The TBI alone and TBI + striatal injury groups showed an increase in hypothalamic NA and 5-HT levels at 3 and 20 days post-injury compared with the sham group (Figures 7A,E). However, cortical NA and 5-HT levels were increased at 3 days on both the ipsilateral and contralateral sides, with the TBI + striatal injury group showing increased serotonergic levels. Both NA and 5-HT are neurotransmitters associated with temperature regulation (Feldberg and Myers, 1963; Clark and Lipton, 1986; Cooper, 2002). For example, NA and 5-HT injection into the hypothalamus decreases temperature and heat production (Cooper et al., 1976; Cox et al., 1980; Lin et al., 1983). These experiments could, in part, explain the increase in hypothalamic NA and 5-HT levels (Kandel et al., 2013) in our study. DA is another neurotransmitter associated with temperature regulation (Cox et al., 1980; Cooper, 2002), and hypothalamic injection of DA decreases core temperature values (Cox et al., 1980). In our results, the hypothalamic DA levels were increased at 20 days post-injury in the TBI + striatal injury group, an effect that could be associated with recovery. However, cortical DA levels were temporarily reduced in the ipsilateral cortex, an effect that may be associated with cortical injury and motor deficits (Shin et al., 2011; Jenkins et al., 2018). In our study, the discrepancies between cortical and hypothalamic DA levels could be explained by the supply source, as the cortex receives mesostriatal and mesocortical dopaminergic input, whereas the hypothalamus receives local dopaminergic input (Björklund and Dunnett, 2007).

In several neurochemical studies using unilateral lesion techniques, both ipsilateral and contralateral monoaminergic effects have been reported (Adèr et al., 1980; Robinson et al., 1980; Krobert et al., 1994; Bueno-Nava et al., 2008, 2010; Ramos-Languren et al., 2016). The bilateral monoaminergic response could result from cortical monoaminergic axonal injury and neurodegeneration after TBI (Hutson et al., 2011; Dougherty et al., 2020). It is well known that efferent axons from monoaminergic nuclei, such as the locus coeruleus, dorsal raphe, and substantia nigra, are highly collateralized in the brain cortex (Takada and Hattori, 1986; Van Bockstaele et al., 1993; Waselus et al., 2011; Chandler et al., 2014; Schwarz and Luo, 2015). These previous findings could explain why an ipsilateral injury may produce changes in cortical NA, DA, and 5-HT levels on the side contralateral to the lesion.

Potential limitations of our study include analyzing neurotransmitter levels in total cortical and hypothalamic tissue, rather than in specific compartments like the extracellular space, where neurotransmitters are actively released. Future experiments should consider the use of microdialysis to target the cortical and hypothalamic areas. For assessing the presence of alpha-2, D_2_, and 5-HT_1_ autoreceptors, immunohistochemical or binding methods should be used. Moreover, using the external acoustic meatus temperature as a proxy for core temperature lacks precision; therefore, implanting intracerebral electrodes may provide more accurate measurements in future research.

In conclusion, our study suggests that TBI with striatal hemorrhagic extension not only influences peripheral and core temperature indicators of autonomic thermoregulatory function but also has a distinct impact on hypothalamic and cortical monoamine levels when compared to TBI alone. The alterations in monoamine levels, specifically NA and 5-HT, are pronounced during both the motor deficit and motor recovery phases and persist in the cortex and hypothalamus for up to 20 days post-injury. The observed changes in hypothalamic monoamines provide indirect evidence of hypothalamic alterations post-injury. Further investigations are essential to correlate cerebral temperature with body temperature following a brain injury.

## Data availability statement

The raw data supporting the conclusions of this article will be made available by the authors, without undue reservation.

## Ethics statement

The animal study was approved by Instituto Nacional de Rehabilitación LGII/CICUAL/011/2020: 91/17, 06/18, and 82/21. The study was conducted in accordance with the local legislation and institutional requirements.

## Author contributions

AV-M: Data curation, Formal analysis, Investigation, Methodology, Project administration, Visualization, Writing – original draft, Writing – review & editing. DM-R: Conceptualization, Investigation, Methodology, Project administration, Software, Supervision, Writing – original draft, Writing – review & editing. SO: Data curation, Investigation, Project administration, Supervision, Writing – original draft. AG-R: Data curation, Investigation, Project administration, Writing – original draft. AA-P: Data curation, Formal analysis, Investigation, Writing – original draft. JC-A: Methodology, Project administration, Supervision, Conceptualization, Writing – original draft. AA-R: Investigation, Supervision, Validation, Writing – review & editing. CP-C: Conceptualization, Formal analysis, Methodology, Software, Writing – review & editing. AA-L: Data curation, Funding acquisition, Investigation, Validation, Writing – original draft, Writing – review & editing. AB-N: Conceptualization, Formal analysis, Funding acquisition, Investigation, Methodology, Supervision, Writing – original draft, Writing – review & editing.
